# High-risk food recalls in the United States: Temporal trends and structural characteristics based on FDA enforcement data, 2012–2024

**DOI:** 10.1371/journal.pone.0356620

**Published:** 2026-08-19

**Authors:** Huiling Chen, Zhichun Xue, Zhaohan Sun, Jia Chen, Qing Xue, Kunhuang Han

**Affiliations:** 1 College of Marine Sciences, Ningde Normal University, Ningde, China; 2 Engineering Research Center of Mindong Aquatic Product Deep-Processing, Ningde, China; 3 Department of Respiratory and Critical Care Medicine, Ningde Clinical Medical College of Fujian Medical University, Ningde, China; 4 Ningde Yiye Marine Industry Development Co., Ltd., Ningde, China; Nitte University, INDIA

## Abstract

**Background:**

Food recalls constitute a central risk management tool within the United States food safety system; however, the distribution of recall severity—particularly high-risk Class I recalls—across temporal and structural dimensions remains insufficiently characterized.

**Methods:**

Food recall data from the Food Enforcement Reports issued by the U.S. Food and Drug Administration (FDA) between 2012 and 2024 were analyzed. In accordance with FDA regulatory definitions, Class I recalls were treated as high-risk outcome events. Recall reasons, food categories, distribution scope, geographic region, and calendar year were systematically classified. Multivariable logistic regression models including recall reason, product category, distribution scope, geographic region, and calendar year (modeled as a categorical variable) were used to evaluate characteristics associated with Class I classification, and marginal standardization was applied to estimate adjusted temporal trends. Exploratory stratified analyses examined Class I proportions across recall reason–food category combinations and over time for the two most frequent recall reasons.

**Results:**

In total, 26,877 FDA-regulated food recall events were included in the analysis. Annual recall counts ranged from approximately 1,100 to more than 3,000 events per year, without evidence of a sustained monotonic trend. Microbial contamination and undeclared allergens accounted for the majority of recall events and were the predominant hazard categories associated with Class I classification. Bacterial pathogen contamination recalls generally showed high Class I proportions across major food categories, whereas undeclared allergen recalls displayed greater variation across product categories and years. Recall classification was closely aligned with hazard-related characteristics, reflecting the regulatory criteria used to define Class I recalls. Classification patterns varied across hazard types. In contrast, no consistent association was observed for distribution scope after adjustment, and associations for product categories and geographic regions were generally modest. No sustained monotonic temporal trend was identified, with substantial inter-annual variability in adjusted probabilities. Sensitivity analyses using alternative outcome definitions and restriction approaches yielded consistent results.

**Conclusions:**

FDA Class I classification patterns in the United States were strongly structured by hazard characteristics, whereas associations with broader structural features, including food categories, geographic regions, and distribution scope, were generally more limited and inconsistent. Exploratory analyses showed relatively stable high Class I proportions across many bacterial pathogen–food profiles but greater variation among allergen-related recalls across product categories and calendar years. These findings characterize patterns in regulatory outcomes and may inform risk-oriented food safety management, but they do not independently establish whether individual FDA classifications were appropriate, proportionate, or scientifically justified.

## 1 Introduction

Food safety has emerged as a major global public health concern, with foodborne diseases imposing a substantial burden on morbidity and mortality worldwide. According to the World Health Organization (WHO), an estimated 600 million people fall ill and 420,000 people die each year as a result of contaminated food, highlighting the critical importance of effective food safety systems. Within this global context, food recall systems represent an essential regulatory tool across jurisdictions, including the United States and the European Union, serving as a key mechanism for risk communication and post-market risk control [1,2].

Food recalls constitute one of the most prominent risk intervention mechanisms in the United States Food Safety Governance System. Their primary function is to facilitate the timely removal of food products from the supply chain when contamination, adulteration, or labeling noncompliance poses a potential threat to human health. Recalls may be initiated voluntarily by industry or mandated through regulatory enforcement, thereby reducing population exposure and mitigating potential public health harm [3,4]. Within a risk-based regulatory framework, food recalls are best understood as a critical component of the food safety risk-management continuum. Specifically, when preventive controls and routine oversight do not fully eliminate residual risks, recalls function as a secondary risk control measure by integrating traceability, risk communication, and corrective actions rather than being interpreted simplistically as evidence of systemic failure [5]. This institutional role is closely aligned with the “prevention-oriented” philosophy emphasized by the Food Safety Modernization Act (FSMA), which advocates allocating regulatory resources based on scientific evidence and risk assessment, as well as deploying regulatory tools, including recalls, when necessary to achieve public health protection objectives [6]. According to the Food and Drug Administration (FDA) recall classification system, food recalls are categorized into Class I, Class II, and Class III, with Class I recalls defined as situations in which there is a reasonable probability that exposure to a product may result in serious adverse health consequences or death [3].

Existing studies have provided an important foundation for understanding the long-term evolution of food recalls in the United States; however, their analytical focus has largely remained descriptive, with primary attention directed toward recall frequency, causal composition, and the distribution of affected product categories. A systematic analysis conducted by the U.S. Department of Agriculture Economic Research Service (USDA ERS), covering the period from 2004 to 2013, reported a substantial increase in the number of food recalls, accompanied by a notable rise in recalls related to undeclared allergens, thereby underscoring the growing importance of labeling compliance within the recall system [7]. From a broader perspective, multiple empirical studies and narrative reviews have consistently identified microbial contamination and labeling or formulation errors as the most prevalent triggers of food recalls. These factors are often associated with inadequate process controls, cross-contamination during production, increasing supply chain complexity, and deficiencies in risk communication mechanisms [8,9]. Nevertheless, despite these contributions, prior research has predominantly focused on how many recalls occurred and why they occurred, while devoting comparatively limited attention to heterogeneity in the severity of potential health consequences across recall events.

At the food category level, different products exhibit distinct risk profiles, owing to variations in raw material composition, processing practices, and consumption patterns. Previous studies have shown that perishable or ready-to-eat foods are more frequently implicated in microbial contamination events, whereas processed foods containing multiple ingredients or produced on shared manufacturing lines face greater challenges in preventing allergen cross-contact [10–16]. Among the various recall triggers, undeclared allergens have particularly important public health implications, as the associated health risks are highly contingent on the completeness and accuracy of the labeling information. Labeling errors are often rooted in systemic vulnerabilities, including inadequate management of formulation changes, insufficient cleaning validation, deficiencies in label review procedures, and gaps in personnel training [17–20]. Regulatory-oriented research further indicates that despite the implementation of allergen-focused legislation such as the Food Allergen Labeling and Consumer Protection Act (FALCPA), allergen-related recalls in the United States have persisted and exhibit substantial variation in scale and structural characteristics across regulatory jurisdictions [21–24]. Collectively, this body of evidence suggests that food category and risk type may play an important role in variation in recall severity; however, the available evidence remains largely descriptive in nature.

Although the FDA recall classification system formally delineates levels of health hazards, recall severity, as reflected by Class I classification, has frequently been treated in the existing literature as a secondary descriptive outcome rather than as a central analytical focus. Under comparable recall counts, Class I recalls differ fundamentally from Class II and Class III recalls with respect to the intensity of potential health outcomes, the size of the population potentially affected, the priority assigned to emergency response, and the associated demands placed on regulatory and public health resources. Consequently, their public health implications are not directly comparable [1,7]. While prior studies have suggested that certain hazard sources, such as undeclared allergens and specific forms of microbial contamination, are more likely to be associated with higher recall classifications in practice [7,22,23], systematic evidence regarding which structural characteristics are most strongly associated with Class I designation remains limited. In particular, factors including food category, hazard type, distribution scope, geographic region, and their evolution over extended time horizons have not been comprehensively evaluated within a unified analytical framework using multivariable approaches [7–9,22–24]. Against the backdrop of increasing supply chain complexity and a continually evolving risk communication environment, reliance on recall counts alone without explicitly incorporating recall severity as a core analytical dimension may yield biased or incomplete assessments of food safety risk trends.

Building on these research gaps, this study systematically characterized FDA food recall events from the perspective of regulatory classification. Using FDA enforcement data from 2012 to 2024, we examined temporal patterns and structural characteristics associated with Class I classification within a multivariable framework. In addition, exploratory stratified analyses were conducted to assess variation in Class I classification across broadly comparable recall profiles defined by recall reason and food product category, and to examine whether classification patterns for the two most frequent recall reasons remained stable over time. Because several variables, particularly recall reason, represent information considered during the FDA classification process, the analyses were intended to characterize patterns in regulatory outcomes rather than to identify independent causal predictors or determine whether individual FDA decisions were scientifically correct or proportionate.

## 2 Methods

### 2.1 Study design and data sources

This study was designed as a retrospective, descriptive analysis based on publicly available regulatory data, with the objective of systematically characterizing temporal trends and structural features of food recall events reported by the U.S. Food and Drug Administration (FDA) between January 1, 2012, and December 31, 2024. Food recall data were obtained from FDA Food Enforcement Reports and accessed via the openFDA platform. The openFDA is an official FDA-maintained structured application programming interface that supports standardized and reproducible data retrieval for regulatory and research purposes. The raw dataset includes information on recall identification numbers, recall initiation dates, recall classification, recall reasons, product descriptions, and distribution scope. The initial dataset encompasses all food-related recall records within the FDA’s regulatory jurisdiction during the study period. The recall event numbers assigned by the FDA were used as unique identifiers to detect and remove duplicate records. After restricting the dataset to the predefined study period and completing the data cleaning procedures, a final analytical dataset was constructed for subsequent statistical analyses.

Data extraction and preprocessing were conducted using a standardized workflow. Food recall records were restricted to FDA-regulated food products (`product_type == “Food”`), and the study period was limited to 2012–2024 based on the recall initiation date. Recall events were deduplicated at the event level using the unique recall identifier (`recall_number`); thus, repeated notices, follow-up updates, or expanded notifications referring to the same recall were counted only once. Both voluntary and mandated recalls were included in the analysis, as both represent regulatory recall events reported in the FDA Enforcement Reports. For recalls involving multi-ingredient products, product categorization was based on the product description field rather than the specific hazard, following predefined rule-based classification procedures. All preprocessing steps were implemented using predefined rules to ensure transparency and reproducibility.

### 2.2 Definition of recall severity

According to the U.S. Food and Drug Administration (FDA), food recalls are classified into three categories based on the potential risk to public health: Class I, Class II, and Class III. Class I recalls are defined as situations in which exposure to a product has a reasonable probability of causing serious adverse health consequences or death. Class II recalls may cause temporary or medically reversible adverse health effects, whereas Class III recalls are unlikely to result in any adverse health effects. For the purpose of this analysis, recall severity was specified as the primary outcome. Class I recalls were designated as severe recall events, while Class II and Class III recalls were combined into non-Class I to focus on recalls with the greatest potential public health impact. Additional sensitivity analyses using alternative outcome definitions, including separate comparisons of Class I versus Class II and Class I versus Class III, are described in Section 3.5 to assess the robustness of the findings.

### 2.3 Classification of recall reasons and product categories

Information on recall reasons was extracted from the recall reason field of the original FDA recall records. To improve interpretability and better capture the heterogeneity of underlying hazards, recall reasons were classified into multiple mutually exclusive categories using a predefined rule-based mapping dictionary (Table S1 in S1 File). These categories included bacterial pathogen contamination, viral contamination, microbial toxin or spore-forming contamination, foreign material contamination, chemical contamination or adulteration, labeling or misbranding (non-allergen), temperature control or storage deviation, regulatory or good manufacturing practice (GMP) deviation, packaging or container defects, spoilage or quality defects, and other or unspecified causes.

To enhance transparency, representative mapping rules were defined a priori. For example, recall descriptions containing terms such as “Salmonella,” “Listeria,” or “E. coli” were classified as bacterial pathogen contamination; references to “metal fragments,” “plastic pieces,” or other extraneous materials were classified as foreign material contamination; and labeling errors not involving allergens were classified under labeling or misbranding. A more comprehensive set of mapping examples is provided in Table S2 in S1 File. This approach illustrates how unstructured textual descriptions were systematically operationalized into structured analytical variables.

Product descriptions in the FDA recall records were recorded as unstructured free-text fields. Product categories were standardized using a combined approach incorporating keyword-based matching rules, characteristic features of FDA product descriptions, and limited manual verification. Categories with small numbers of recall events were consolidated to reduce data sparsity and improve model stability. The detailed classification criteria for product categories are provided in Table S3 in S1 File.

The classification procedure was primarily rule-based and reproducible. Manual review was performed only in a limited number of ambiguous cases to ensure consistency with predefined rules.

### 2.4 Definition of distribution scope and geographic variables

Based on the distribution information disclosed in FDA recall records, recall events were categorized into four primary distribution scopes: single-state (local), multi-state, nationwide, and international. Records with missing or unspecified distribution information were classified as “unknown/other” and included as a separate category in the analysis. The geographic region was defined based on the location of the recalling firm as reported in the FDA recall records. Specifically, the firm location field was used to assign each recall event to one of four U.S. Census regions: Northeast, Midwest, South, and West. Records with missing or ambiguous location information were categorized as “unknown”.

### 2.5 Statistical analysis

Descriptive statistical analyses were conducted to summarize the overall characteristics of food recall events, including annual recall counts, proportional distribution of recall classifications, and distributions of recall reasons, product categories, distribution scope, and geographic region. Categorical variables are summarized as frequencies and percentages. To quantify variation in observed FDA Class I classification across recall characteristics, multivariable logistic regression models were fitted with recall classification specified as a binary outcome (Class I vs non-Class I). Although FDA recall classification comprises three ordered categories, the primary analysis used a binary outcome because the prespecified scientific objective was to distinguish Class I recalls—the category representing the highest potential public health consequence—from all other recall classifications. An ordinal logistic model was not selected as the primary approach because the study did not aim to estimate a single proportional shift across all three classification levels, and the proportional-odds assumption may not adequately represent potentially different contrasts between Class I, Class II, and Class III recalls. To assess whether combining Class II and Class III influenced the findings, separate Class I versus Class II and Class I versus Class III sensitivity analyses were conducted. The explanatory characteristics included product category, recall reason, distribution scope, geographic region, and calendar year. The calendar year was modeled as a categorical variable to account for potential non-linear temporal trends. To account for potential clustering of recall events within firms, cluster-robust standard errors were applied at the firm level. Regression results are reported as ORs with corresponding 95% CIs. Model specification was defined a priori based on subject-matter relevance, and all covariates were included simultaneously in the multivariable models without stepwise selection. Potential multicollinearity was considered in model construction, and no substantial collinearity was evident among included variables. Interaction terms were not included in the primary models, as the study aimed to provide an overall descriptive assessment rather than to evaluate effect modification. Model fit was evaluated using standard logistic regression diagnostics. Missing data in key variables were handled by assigning separate categories (e.g., “unknown” or “unspecified”) to preserve sample size and avoid selection bias. Although the data span multiple years, the analysis was conducted at the recall-event level rather than as a longitudinal panel. More complex time-series or mixed-effects models were not applied given the descriptive objective of the study. To evaluate the robustness of the findings, additional analyses were conducted using alternative outcome definitions (Class I vs Class II and Class I vs Class III), as well as a restriction analysis retaining only one recall per firm per year. Within this framework, variables included in the analysis can be conceptually distinguished into different roles. Recall reason and distribution scope are considered characteristics that may reflect information available to regulators during the FDA classification process and thus can be viewed as components or inputs to classification. In contrast, variables such as product category and geographic region may represent broader contextual or structural features of the food system.

### 2.6 Temporal trend analysis

To characterize temporal variation in the probability of Class I recalls, we estimated adjusted predicted probabilities for each year using multivariable logistic regression models. The models included recall reason, product category, distribution scope, and geographic region as covariates, with calendar year modeled as a categorical variable. Adjusted probabilities were derived using marginal standardization, whereby predicted probabilities were calculated for each observation while setting the year to a specific level and then averaged across the study population.

### 2.7 Exploratory analysis of variation in regulatory classification patterns

To explore whether recalls with broadly comparable observable characteristics received similar regulatory classifications, we calculated the proportion of Class I recalls within strata jointly defined by recall reason category and food product category. Class II and Class III recalls were combined as non-Class I events, consistent with the primary outcome definition. To reduce instability arising from sparse cells, interpretation and visualization were restricted to recall reason–food category combinations containing at least 20 events.

Temporal variation was additionally examined for the two most frequent recall reason categories, bacterial pathogen contamination and undeclared allergen. For each calendar year, the observed number and proportion of Class I recalls were calculated separately for these two categories.

These analyses were exploratory and descriptive. They were designed to evaluate variation in observed classification patterns across comparable recall profiles and over time, but not to determine whether individual classifications were correct, scientifically justified, proportionate, or erroneous, because the public dataset did not contain all information used in FDA health-hazard evaluations.

### 2.8 Sensitivity analyses

To evaluate the robustness of the main findings, several sensitivity analyses were conducted. First, to address potential information loss arising from the binary outcome specification, alternative outcome definitions were applied. Specifically, two additional logistic regression models were constructed: (1) Class I versus Class II recalls and (2) Class I versus Class III recalls. These analyses were conducted to assess whether the observed associations were sensitive to different severity contrasts. Second, to account for clustering of recall events within firms, cluster-robust standard errors were applied at the firm level in the main analysis. In addition, a restriction analysis was conducted by retaining only one recall event per firm per year, thereby minimizing the potential influence of repeated recalls from the same firm. All sensitivity analyses were specified using the same set of covariates as the main model, including recall reason category, product category, distribution scope, geographic region, and calendar year. Results are reported as ORs with corresponding 95% CIs.

### 2.9 Software and reproducibility

All data processing, statistical analyses, and figure generation were performed using the R software (version 4.3). All statistical tests were two-sided, and a significance threshold of 0.05 was applied. Detailed data processing workflows, variable construction procedures, and analysis codes are provided in the Supplementary Materials to ensure transparency and reproducibility of the study.

## 3 Results

### 3.1 Overall characteristics of FDA food recall events in the United States, 2012–2024

**Fig 1:**
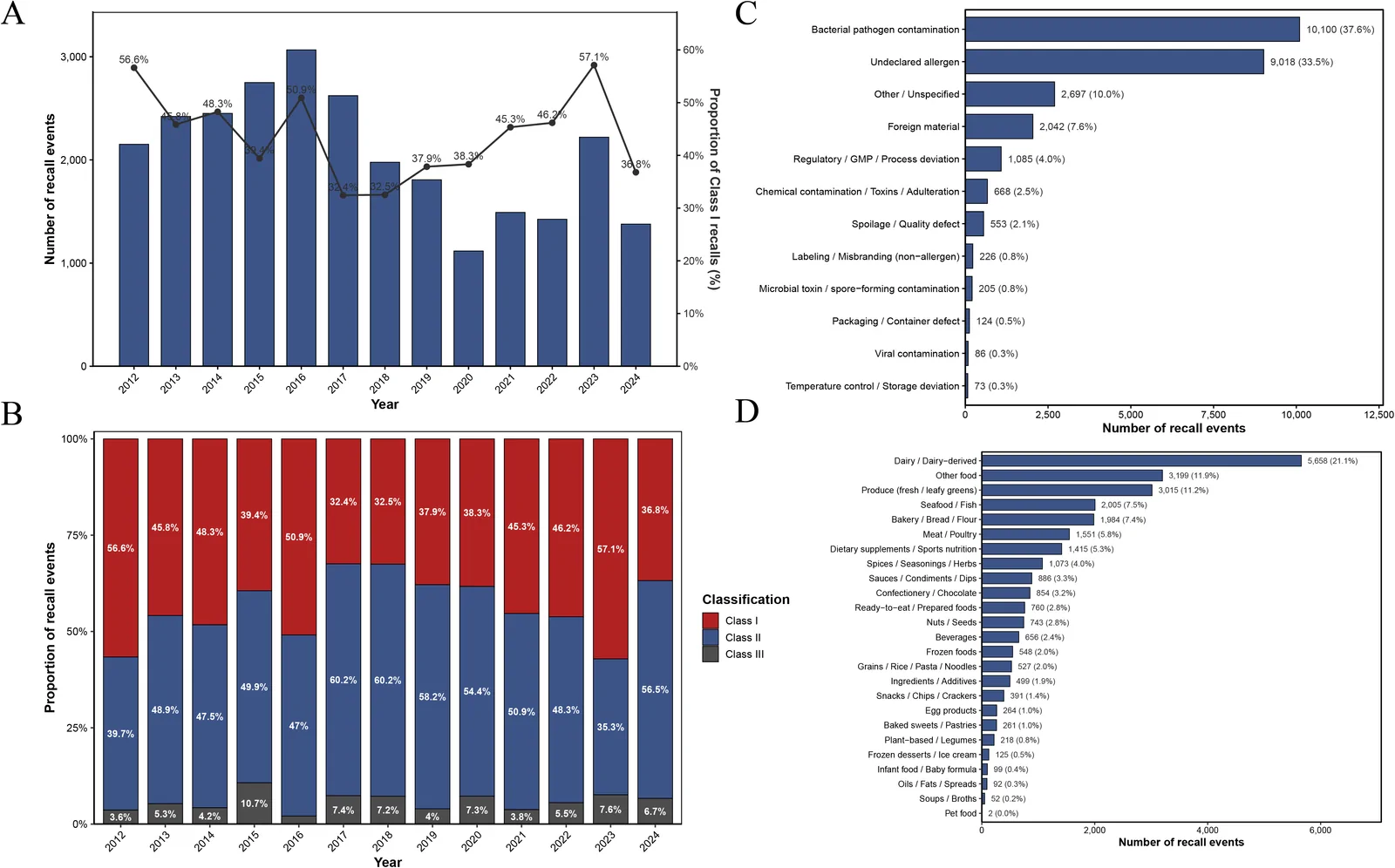
Overview of FDA food recall events in the United States from 2012 to 2024. a, Annual number of FDA-regulated food recall events. b, Yearly distribution of recall classifications (Class I, Class II, and Class III), presented as 100% stacked bars. c, Overall distribution of recall reasons grouped into major categories. d, Overall distribution of recalled food product categories.

Between January 1, 2012, and December 31, 2024, 26,877 human food recall events regulated by the U.S. FDA were included in the final analytical dataset (Table S4 in S1 File). Annual recall counts varied substantially over the study period (Fig 1A; Table S4 in S1 File), ranging from 1,117 events in 2020–3,066 events in 2016. Overall, recall frequency increased between 2012 and 2016, declined markedly from 2017 to 2020, and then partially rebounded, with considerable year-to-year fluctuation and no sustained monotonic long-term pattern.

With respect to recall classification, Class II recalls were the most frequent category overall, accounting for 13,452 events (50.1%), followed by Class I recalls with 11,871 events (44.2%) and Class III recalls with 1,554 events (5.8%) (Fig 1B; Table S4 in S1 File). Across individual years, the proportion of Class I recalls varied substantially, whereas Class III recalls consistently represented a relatively small share of all recalls (Fig 1A).

Regarding recall reasons, bacterial pathogen contamination was the most common category, accounting for 10,100 events (37.6%), followed by undeclared allergen with 9,018 events (33.6%) (Fig 1C; Table S4 in S1 File). Foreign material accounted for 2,042 events (7.6%), and regulatory or good manufacturing practice (GMP) or process deviations accounted for 1,085 events (4.0%). All remaining reason categories individually accounted for less than 3% of all recall events.

Across product categories, recall events were distributed unevenly across food types (Fig 1D; Table S4 in S1 File). Dairy and dairy-derived products were the most frequently recalled category, accounting for 5,658 events (21.1%), followed by other food (11.9%) and produce, including fresh or leafy greens (11.2%). Seafood or fish, bakery, bread or flour products, meat or poultry, and dietary supplements or sports nutrition products each accounted for approximately 5%–8% of all recalls.

### 3.2 Structural distribution of recall reasons across food categories

**Fig 2:**
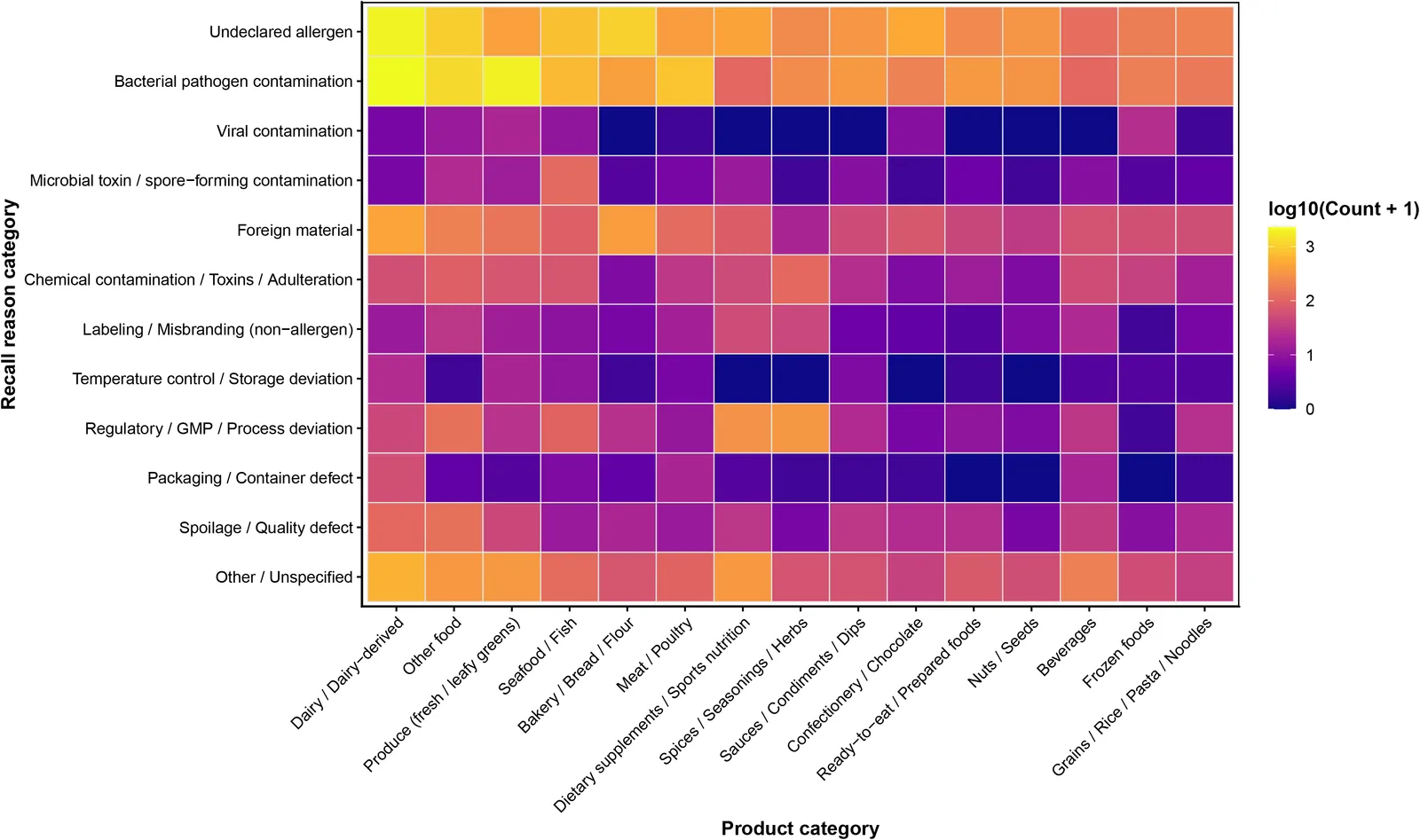
Heatmap of recall reasons by food product category among the most frequently recalled products, 2012–2024. The heatmap depicts co-occurrence patterns between grouped recall reasons and food product categories among the 15 most frequently recalled product categories. Color intensity represents the log-transformed number of recall events (log₁₀[count + 1]), with darker shading indicating higher frequencies.

To characterize the distribution of recall reasons across food categories, we examined the joint distribution of recall reasons and product categories and visualized it using a heat map (Fig 2). The analysis highlights the 15 food categories with the highest recall frequencies, while detailed results for lower-frequency categories are provided in the Supplementary Materials (Table S5 in S1 File).

Microbial contamination, including bacterial pathogens and microbial toxins, was most commonly observed in dairy and dairy-derived products, fresh produce (including leafy greens), meat and poultry, seafood, and ready-to-eat/prepared foods. Undeclared allergens were most frequently observed in bakery products, dietary supplements, condiments and sauces, confectionery/chocolate, and nuts/seeds. Foreign material contamination was most commonly observed in dairy products, bakery products, and other processed food categories. Recalls attributed to regulatory or GMP deviations were more frequently observed in spices, dietary supplements, and other processed food categories. Chemical contamination/adulteration, spoilage/quality defects, packaging/container defects, labeling/misbranding (non-allergen), viral contamination, and temperature control/storage deviations were generally less frequent across most food categories.

### 3.3 Associations between recall characteristics and Class I classification

**Fig 3:**
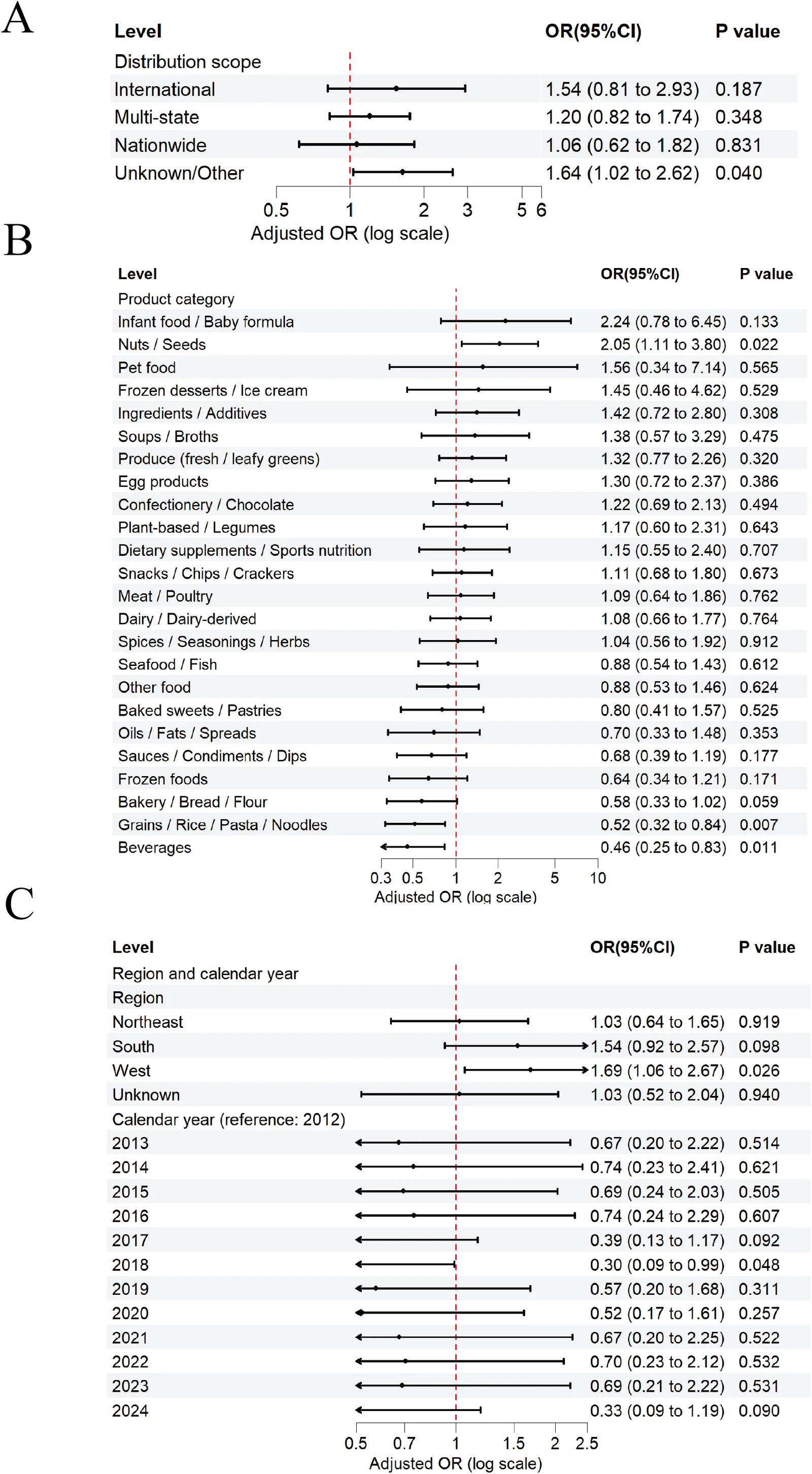
Multivariable-adjusted associations between recall characteristics and Class I food recalls. Forest plots show adjusted odds ratios (aORs) and 95% confidence intervals for associations between recall characteristics and Class I classification. A. Distribution scope, with single-state recalls as the reference category. B. Food product categories, with ready-to-eat/prepared foods as the reference category. C. Geographic region and calendar year, with the Midwest region and calendar year 2012 as the reference categories.

Multivariable logistic regression models were used to quantify associations of recall reason, distribution scope, product category, geographic region, and calendar year with observed Class I classification. All characteristics were mutually adjusted, and the results are presented as adjusted odds ratios (aORs) with corresponding 95% confidence intervals (Fig 3; Table S6 in S1 File).

With respect to distribution scope, no statistically significant associations were observed for international (aOR = 1.54, 95% CI: 0.81–2.93; P = 0.187), multi-state (aOR = 1.20, 95% CI: 0.82–1.74; P = 0.348), or nationwide recalls (aOR = 1.06, 95% CI: 0.62–1.82; P = 0.831), compared with single-state recalls. However, recalls with unknown or other distribution categories were associated with higher odds of Class I classification (aOR = 1.64, 95% CI: 1.02–2.62; P = 0.040) (Fig 3A).

At the product category level, most categories were not significantly associated with Class I classification. Nuts and seeds were associated with higher odds (aOR = 2.05, 95% CI: 1.11–3.80; P = 0.022). In contrast, grains, rice, pasta, and noodles (aOR = 0.52, 95% CI: 0.32–0.84; P = 0.007) and beverages (aOR = 0.46, 95% CI: 0.25–0.83; P = 0.011) were associated with lower odds of Class I classification. Other product categories were not statistically significant (Fig 3B).

In the geographic analysis, recalls from the West region were associated with higher odds of Class I classification (aOR = 1.69, 95% CI: 1.06–2.67; P = 0.026), whereas no statistically significant associations were observed for the Northeast, South, or unknown regions (Fig 3C).

When calendar year was modeled as a categorical variable, the adjusted odds of Class I recalls varied across years without a consistent monotonic trend. Compared with 2012, only 2018 showed a statistically significant difference (aOR = 0.30, 95% CI: 0.09–0.99; P = 0.048), while other years were not significantly different.

### 3.4 Adjusted temporal trends in high-risk food recalls

**Fig 4:**
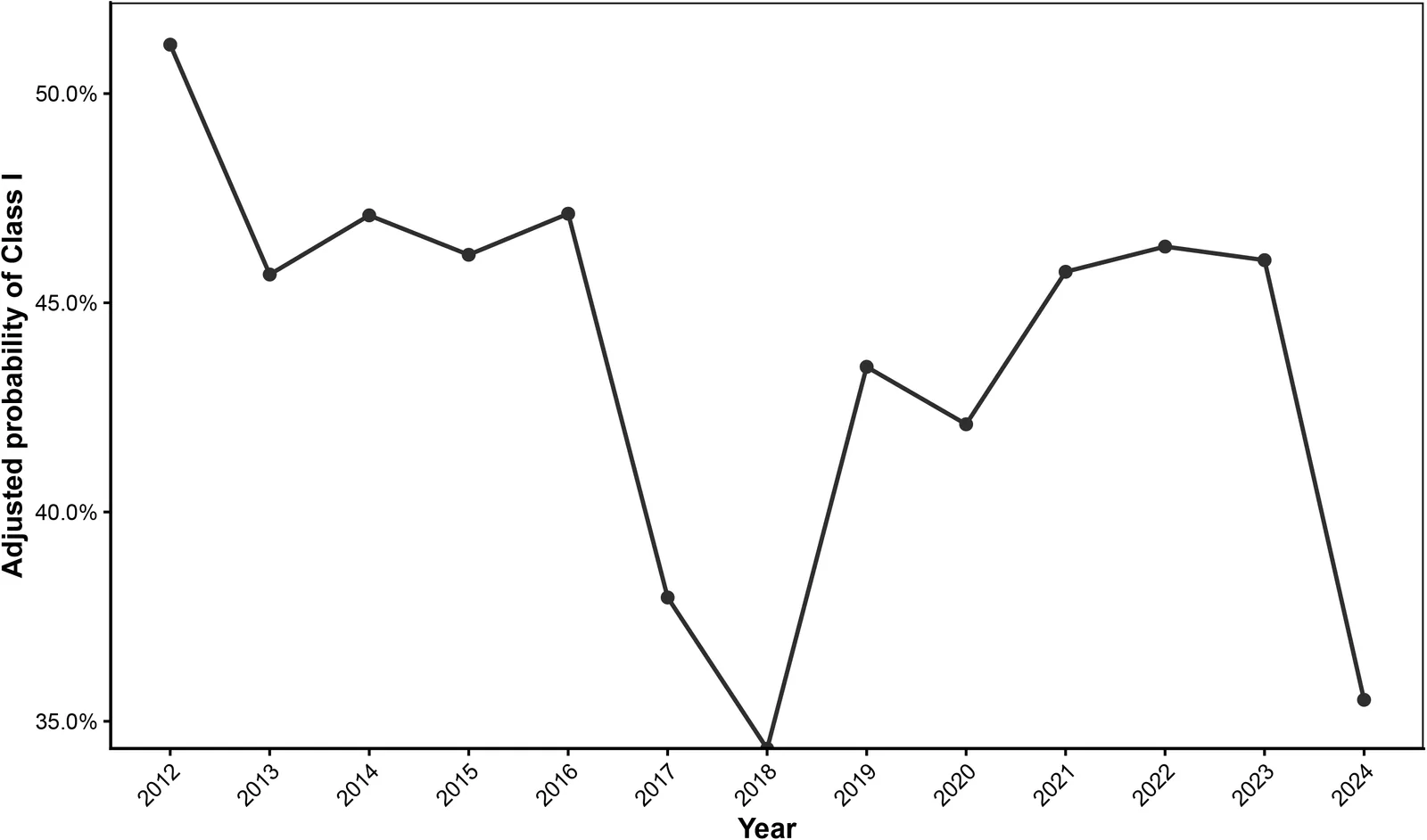
Adjusted temporal trends in high-risk food recalls in the United States, 2012–2024. Adjusted predicted probability of Class I food recalls by calendar year, estimated from a multivariable logistic regression model using marginal standardization and adjusting for recall reason, product category, distribution scope, and geographic region.

Based on multivariable logistic regression models, adjusted predicted probabilities of Class I (high-risk) food recalls were estimated using marginal standardization, adjusting for recall reason, product category, distribution scope, and geographic region. As shown in Fig 4 and Table S7 in S1 File, the adjusted probability of Class I classification varied across calendar years between 2012 and 2024, without evidence of a consistent monotonic increasing or decreasing trend. Higher probabilities were observed in the early study period, followed by a marked decline around 2017–2018, and subsequent fluctuations in later years. In particular, the adjusted probability reached its lowest level in 2018, after which it rebounded and remained relatively stable between 2019 and 2023, before declining again in 2024.

### 3.5 Exploratory variation in Class I classification across comparable recall profiles

Exploratory stratified analyses showed structured variation in Class I classification across recall reason–food category combinations. Among bacterial pathogen contamination recalls, Class I proportions were consistently high across several major food categories, including dairy and dairy-derived products (84.3%), fresh produce (85.0%), meat or poultry (83.1%), and seafood or fish (81.9%). In contrast, undeclared allergen recalls showed greater variation across food categories. The Class I proportion was 20.5% for bakery, bread, or flour products, 34.1% for seafood or fish, 43.8% for dairy and dairy-derived products, 44.5% for dietary supplements or sports nutrition products, and 48.5% for fresh produce (Supplementary Table S9 in S1 File; Supplementary Fig S1 in S2 File).

Temporal patterns also differed between the two most frequent recall reasons. For bacterial pathogen contamination, the annual Class I proportion was generally high but fluctuated from 55.4% to 92.3% across the study period. Undeclared allergen recalls showed lower and more variable annual Class I proportions, ranging from 20.7% to 64.4%. Neither category exhibited a sustained monotonic increase or decrease over time (Supplementary Table S10 in S1 File).

### 3.6 Sensitivity analyses

The results of the sensitivity analyses were broadly consistent with those of the main analysis (Table S8 in S1 File). When alternative outcome definitions were applied (Class I vs Class II, Class I vs Class III) and in the restriction analysis retaining one recall per firm per year, the overall pattern of associations among product categories, distribution scope, geographic region, and calendar year remained generally consistent in direction and magnitude.

## 4 Discussion

Using publicly available FDA food recall data from 2012 to 2024, this study characterized temporal and structural patterns in Class I regulatory classification. Recall reason showed the strongest relationship with Class I designation, whereas most product-category, distribution-scope, and geographic associations were modest or imprecise after adjustment. Several subgroup estimates had wide confidence intervals, indicating limited precision despite the large overall sample size; these estimates should therefore be interpreted cautiously. Exploratory analyses added further resolution by showing that bacterial pathogen contamination recalls generally maintained high Class I proportions across major food categories, whereas undeclared allergen recalls exhibited greater variation across product categories and calendar years. These findings indicate that FDA classification outcomes are strongly structured by hazard characteristics but are not completely uniform within broad hazard categories. Because recall reason and hazard type are themselves central components of FDA health-hazard evaluation, their strong associations with Class I designation are expected. The regression models should therefore be interpreted as quantifying the structure of FDA classification outcomes rather than identifying independent predictors of intrinsic food safety risk or reproducing an external causal risk model.

From a regulatory surveillance perspective, the findings illustrate how observed Class I classifications are distributed across hazard-related and broader structural characteristics in publicly reported FDA recall records. The results may support descriptive monitoring, communication of recall patterns, and identification of product–hazard profiles warranting further event-level review. However, because hazard-related variables are integral to FDA health-hazard evaluation, these associations should not be used alone to establish surveillance priorities or infer intrinsic differences in food safety risk.

No consistent association was observed between broader distribution scope and Class I classification after multivariable adjustment. This association should not be interpreted as an indication that a wider distribution directly increases the intrinsic hazard of a food product. Rather, it plausibly reflects the composite informational role of the distribution scope within the regulatory framework. According to the institutional definition of Class I recalls issued by the FDA, classification is determined not only by hazard severity but also by the potential consequences of exposure, size of the affected population, and complexity of incident management [3]. Within comparable product categories and regional contexts, recalls involving broader distribution typically entail greater potential population exposure, more complex traceability requirements, and more intensive risk communication demands, all of which may be considered in regulatory decision-making processes. Accordingly, the distribution scope is best interpreted as a composite proxy indicator associated with Class I classification, rather than as a direct measure of intrinsic hazard severity.

After adjusting for distribution scope, geographic region, and temporal factors, a limited number of food categories remained associated with Class I recall classification, indicating that higher recall frequency does not necessarily correspond to a greater probability of high-risk classification. This pattern may reflect differences in hazard profiles and exposure characteristics across food categories. For example, biological pathogens and undeclared allergens, which can lead to severe adverse outcomes at relatively low exposure levels, may be more likely to meet high-risk classification criteria [25,26]. In addition, food categories involving complex processing workflows, multi-ingredient formulations, or shared production lines may be more prone to microbial or allergen cross-contact. Ready-to-eat foods, which offer limited opportunities for exposure mitigation at the point of consumption, may also be subject to more rapid regulatory escalation during risk assessment. These considerations are intended to aid in the interpretation of the observed associations and do not imply that any specific food category is intrinsically more hazardous. Moreover, factors such as regulatory sampling strategies, intensity of industry self-testing, production scale, and sectoral concentration may influence the likelihood that different food categories enter the recall system, thereby shaping the risk distributions observed in regulatory data. Because information on production volume, product market share, inspection frequency, laboratory testing intensity, and industry self-testing was unavailable, the observed differences cannot distinguish variation in underlying hazard risk from variation in market exposure or opportunities for detection.

A limited geographic association with Class I classification was observed, but this finding should be interpreted cautiously because only one regional estimate reached statistical significance and several potentially important event-level determinants of FDA classification were unavailable. The observed regional difference may therefore reflect unmeasured variation in product characteristics, affected consumer populations, hazard concentration, epidemiological evidence, inspection practices, or industry structure rather than an independent geographic effect. Along the temporal dimension, no sustained monotonic trend in Class I classification was identified; instead, year-specific adjusted probabilities showed marked inter-annual variation. Temporal variation in Class I classification should not be interpreted as direct evidence of changes in underlying food safety risk. During the study period, food safety surveillance, laboratory sensitivity, and the use of whole-genome sequencing expanded, while implementation of the Food Safety Modernization Act and related preventive-control practices matured. These developments may have altered hazard detection, evidence accumulation, outbreak linkage, and regulatory decision-making. Changes in the annual composition of recalled hazards and products may also have influenced the observed Class I proportions. Therefore, the temporal patterns reported here likely reflect a combination of surveillance, detection, regulatory, and market processes rather than changes in intrinsic food safety risk alone. Similar patterns, characterized by gradual long-term changes coexisting with pronounced short-term fluctuations, have been reported in previous analyses of U.S. food recall trends [7,27,28].

The exploratory stratified analyses provide a preliminary assessment of variation in regulatory classification among recalls sharing broad observable characteristics. High Class I proportions across multiple bacterial pathogen–food profiles suggest relatively stable classification patterns for many microbiological recalls. In contrast, the wider variation observed among undeclared allergen recalls indicates that classification may depend on additional event-specific considerations not captured by broad hazard and product categories. These may include the specific allergen involved, concentration, intended consumer group, likelihood of exposure, epidemiological evidence, distribution quantity, traceability, and the availability of corrective actions. However, variation within a recall reason–food category stratum should not be interpreted as evidence of regulatory inconsistency or misclassification. Events grouped within the same broad profile may differ materially in exposure probability, expected clinical consequences, affected populations, and evidence available to regulators. Accordingly, the present analysis can identify areas of classification variation but cannot independently determine whether FDA decisions were proportionate, scientifically justified, or erroneous.

This study had several limitations. First, data derived from openFDA and the FDA Enforcement Reports represent regulatory recall events that were detected and publicly reported rather than the underlying incidence or rate of food safety hazards. The dataset did not include appropriate denominator information, including product-specific production volume, market share, or population consumption. It also lacked systematic measures of inspection frequency, laboratory testing intensity, and industry self-testing. Consequently, differences in recall counts or Class I proportions across product categories, regions, or calendar years may reflect variation in production scale, market exposure, surveillance intensity, and opportunities for hazard detection rather than differences in underlying food safety risk. The findings should therefore be interpreted as patterns among documented FDA recall events and not as incidence rates or comparative measures of intrinsic product risk. Second, recall classification may be influenced by the accumulation of evidence over time, advances in traceability, and evolving risk communication practices, introducing a degree of path dependence in regulatory decision-making. In addition, some variables included in the analysis, particularly recall reason and hazard-related characteristics, are themselves central components of FDA health-hazard evaluation. Their strong associations with Class I designation are therefore expected and partly reflect the structure of the FDA classification framework. Accordingly, these associations should be interpreted as descriptive features of observed regulatory classification outcomes rather than as independent external predictors or causal determinants of intrinsic food safety risk. Distribution scope may likewise represent information available during the classification process and should be interpreted cautiously. Third, although multiple covariates were included in the models, residual confounding from unmeasured factors (e.g., firm-level practices, inspection intensity, and reporting behaviors) cannot be excluded. Moreover, while clustering of recall events within firms was addressed using cluster-robust standard errors and sensitivity analyses, residual within-firm correlation or temporal dependence may not be fully captured. In addition, some subgroup estimates were based on relatively sparse data and had wide confidence intervals, limiting precision. Fourth, the exploratory classification-pattern analyses grouped events according to broad observable characteristics and could not establish that events within each stratum were clinically or regulatorily equivalent. The dataset lacked several determinants potentially central to FDA classification, including vulnerable consumer groups, ready-to-eat status, intended consumers, pathogen or allergen concentration, epidemiological linkage to illness, outbreak association, traceability effectiveness, and quantity distributed. These event-level characteristics may play a more direct role in FDA health-hazard evaluation than broader contextual variables such as geographic region. Their absence may have resulted in residual confounding and limits the interpretation of the adjusted associations. Consequently, the observed geographic differences should not be interpreted as independent regional effects, and the study cannot determine whether apparently different classifications among broadly similar events represent appropriate case-specific decisions or regulatory inconsistency. Future studies integrating production volume, product market share, consumption data, inspection frequency, laboratory testing intensity, industry self-testing, outbreak surveillance, and other exposure-related denominators may help distinguish underlying food safety risk from differences in detection opportunity and regulatory surveillance.

## 5 Conclusions

In summary, FDA Class I classification patterns were strongly structured by hazard-related characteristics embedded within the regulatory framework, whereas associations with product category, geographic region, and distribution scope were generally limited or inconsistent. Exploratory analyses showed relatively stable high Class I proportions across many bacterial pathogen–food profiles but greater variation among undeclared allergen recalls across product categories and calendar years. These findings characterize documented regulatory outcomes rather than independent predictors of intrinsic food safety risk and do not establish whether individual FDA classifications were appropriate or proportionate.

## Supporting information

S1 File Supplementary tables S1–S10. (XLSX)

S2 File Supplementary figure S1. Variation in FDA Class I classification proportions across recall reason–food category combinations. (TIF)
